# A bibliometric analysis of metabolic dysfunction-associated steatotic liver disease in children from 2004 to 2024

**DOI:** 10.3389/fped.2025.1468788

**Published:** 2025-04-28

**Authors:** Xiaowei Gong, Siyu Bai, Enze Lei, Tao Lu, Yao Chen, Jianxin Cai, Jianzhong Liu

**Affiliations:** ^1^ College of Traditional Chinese Medicine, Hubei University of Chinese Medicine, Wuhan, China; ^2^ Department of Pediatrics, Wuhan Hospital of Traditional Chinese Medicine, Wuhan, China; ^3^ Department of Pediatrics, Hubei Provincial Hospital of Traditional Chinese Medicine, Affiliated Hospital of Hubei University of Chinese Medicine, Wuhan, China; ^4^ Hubei Shizhen Laboratory, Wuhan, China; ^5^ Hubei Key Laboratory of Liver and Kidney Research and Application of Traditional Chinese Medicine, Wuhan, China

**Keywords:** metabolic dysfunction-associated steatotic liver disease, children, bibliometric, visual analysis, VOSviewer, CiteSpace

## Abstract

**Background:**

Metabolic dysfunction-associated steatotic liver disease (MASLD), once known as Non-alcoholic fatty liver disease, impacts between 3% and 10% of children and adolescents globally, as well as nearly one-third of obsessed boys and one-quarter of obsessed girls, and is the most frequent cause of pediatric liver disease associated with the obesity epidemic. With the growing attention and increasing volume of literature on pediatric MASLD, there is an urgent need for bibliometric analysis and visualization in the area of pediatric MASLD study in terms of dissecting study priorities.

**Methods:**

Literature was searched in the Web of Science Core Collection database, followed by categorization, bibliometric study as well as visual analysis conducted by applying software including Citespace, VOSviewer, and the R language. The study concentrated on analyzing information related to key authors, spatial and temporal distribution, core keywords, and important citations.

**Results:**

In total, 3,409 publications on pediatric MASLD were collected in the study, including 2,697 articles and 712 review articles. Between 2004 and 2024, the volume of publications had been constantly increasing per year. The country with the most numerous publications was the United States, which had extensive exchanges and collaborations with Italy, China, and England, followed by Italy. The *Journal of Pediatric Gastroenterology and Nutrition* had the greatest quantity of publications in this domain. The core literature was a clinical guideline. Insulin resistance, metabolic syndrome, steatohepatitis, hepatocellular carcinoma, cardiovascular risk, diabetes risk, diagnostic accuracy, lifestyle intervention, gut microbiome, probiotics, and metabolic dysfunction-associated steatotic liver disease were also hot topics and frontier trends in pediatric MASLD studies.

**Conclusion:**

This research represents the inaugural application of bibliometric analysis to examine the developmental trajectory of pediatric MASLD studies over the past two decades, which reveals that the etiology, pathological changes of the liver, relationship with obesity, complications, comorbidities, diagnosis and treatments of pediatric MASLD are the key focuses and provides academic references for pediatric clinicians and scholars to grasp the hotspots, the cutting edge and the evolving trends in the area.

## 1 Introduction

Non-alcoholic fatty liver disease (NAFLD) is a clinical liver syndrome featuring hepatocellular steatosis, which is a chronic progressive liver disease that is very common in adults (1). With the increasing prevalence of obesity globally, NAFLD demonstrates a notable prevalence in younger age groups, including children and adolescents (2), which has emerged as the most prevalent etiology of chronic liver disease (CLD) in children (3). According to some studies, the international prevalence of NAFLD in children and adolescents ranges from 3% to 10% (4). Recent studies have estimated that almost one-third of males and one-quarter of females among obese children are affected by NAFLD (5). In the United States, 24.2% of adolescents suffer from a moderate level of hepatic steatosis, and 4.4% suffer from severe hepatic fibrosis; some may even require liver transplantation (6). As a presentation of hepatic metabolic syndrome, NAFLD is firmly associated with a multitude of metabolic diseases, chronic kidney disease, and cardiovascular risks (7). Since 2020, some scholars have recommended reformulating and redefining NAFLD as metabolism-associated fatty liver disease (MAFLD) (8), nevertheless, the terms NAFLD and MAFLD are now superseded, as numerous experts and organizations have endorsed the 2023 proposal of the nomenclature “metabolic dysfunction-associated steatotic liver disease” (MASLD) as being most consistent with the pathologic features of the disease (9). Distinguished from adults, MASLD in children has unique etiological and pathological characteristics as well as therapeutic approaches (10). The etiology, pathology, and treatments of pediatric MASLD require further research.

At present, scholars have conducted a significant number of clinical and experimental studies on MASLD in children (11), and the amount of publications has increased annually. Still, it is challenging to capture the latest trends and hotspots. In comparison, studies such as reviews and meta-analyses are unable to provide forecasting of research trends and visualization of analyses (12). As a specialized field within information science, bibliometrics has evolved over a span of nearly six decades. It has significant application significance in scientific research assessment, discipline development, and academic communication (13), which can help scholars gain a deeper insight into the laws and trends of the academic landscape, and promote the development of academic research and communication (14). There are currently a considerable number of bibliometric studies on obesity and MASLD in adults (15). Based on the existing literature, no bibliometric studies on MASLD in children have been published. With the growing volume of research in the field of MASLD in pediatric and adolescent populations worldwide, along with the existence of more issues to be resolved (16), there is an urgent demand to summarize the current hotspots and trends of research on MASLD in children, which will serve as a reference for more scholars to conduct research in this field in the future.

## 2 Materials and methods

### 2.1 Data source and search strategy

**Figure 1:**
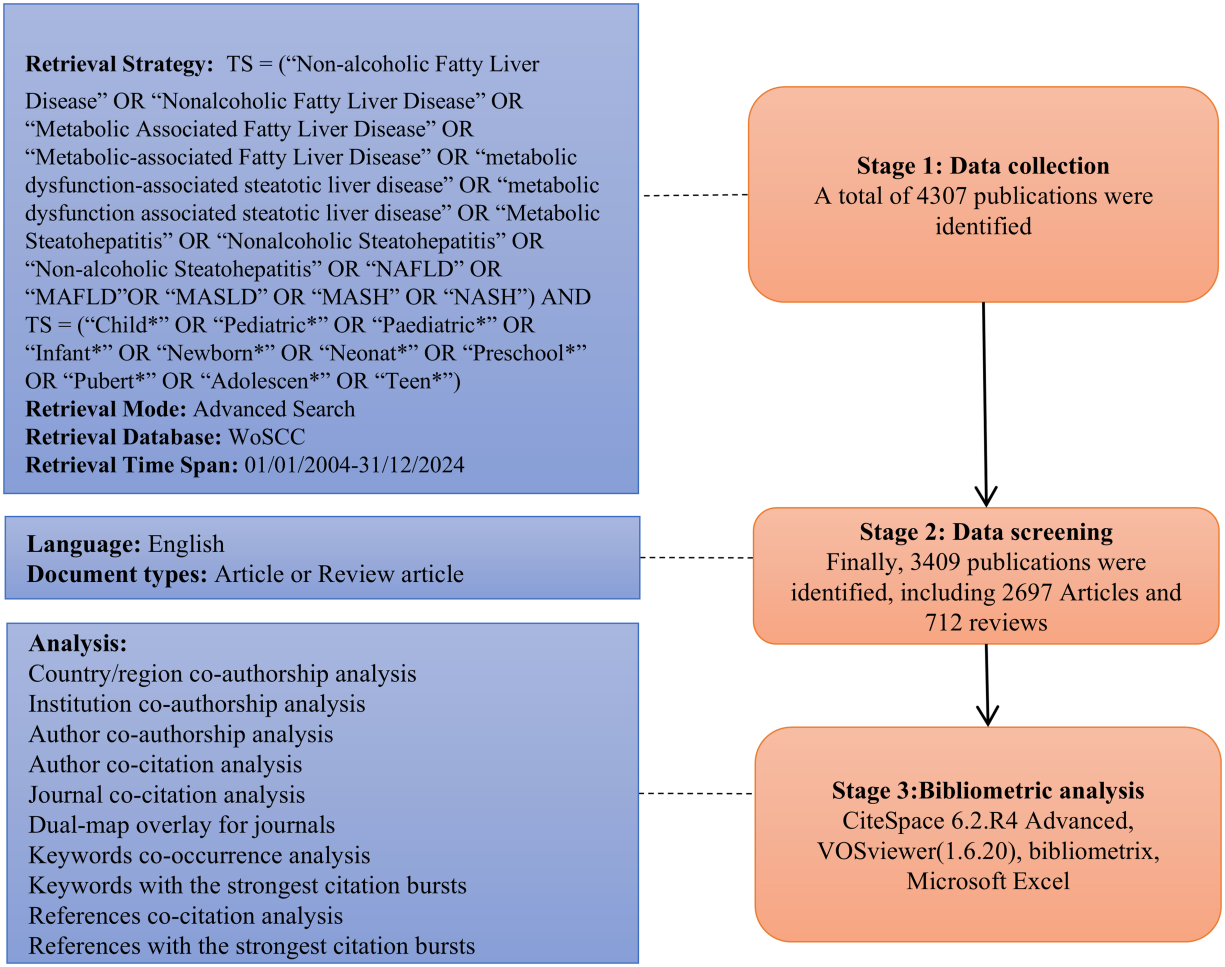
Flow chart of publication retrieval and visual analysis. TS is the abbreviation for topic search, and WoSCC is the abbreviation for the web of science core collection database.

The Web of Science (WOS) database, developed and maintained by Clarivate Analytics (Philadelphia, PA, USA), was selected for data collection owing to its widespread use and suitability for bibliometric analysis (17). This study applied a subject term search methodology to conduct a literature search in the Web of Science Core Collection database (WoSCC). Based on searching the Medical Subject Headings (MeSH) and related literature (15, 18, 19), the search formula was set as TS = (“Non-alcoholic Fatty Liver Disease” OR “Nonalcoholic Fatty Liver Disease” OR “Metabolic Associated Fatty Liver Disease” OR “Metabolic-associated Fatty Liver Disease” OR “metabolic dysfunction-associated steatotic liver disease” OR “metabolic dysfunction associated steatotic liver disease” OR “Metabolic Steatohepatitis” OR “Nonalcoholic Steatohepatitis” OR “Non-alcoholic Steatohepatitis” OR “NAFLD” OR “MAFLD” OR “MASLD” OR “MASH” OR “NASH”) AND TS = (“Child*” OR “Pediatric*” OR “Paediatric*” OR “Infant*” OR “Newborn*” OR “Neonat*” OR “Preschool*” OR “Pubert*” OR “Adolescen*” OR “Teen*”). Within them, “Topic Search” was indicated by “TS”. The search criteria were identified as follows: the search date was restricted to January 1, 2004 to December 31, 2024; only English was accepted in terms of language; the document types were limited to articles or review articles; the search results were exported as plain-text files with customized fields, comprising authors, titles, sources, abstracts, keywords, institutional addresses, cited references, funding information, and other related information. The specific literature search procedure is illustrated in Figure 1.

### 2.2 Data analysis and visualization

VOSviewer is a software program designed by Nees Jan van Eck and Ludo Waltman to visualize the development of collaborations and themes in the field of research. In this study, the VOSviewer (version 1.6.20) software, developed by the Centre for Science and Technology Studies (CWTS) at Leiden University, the Netherlands, was used to conduct the following analyses: distribution of countries/regions, institutions, authors, journals, keywords, references, as well as collaborative analyses in the area of pediatric MASLD research. Citespace is a bibliometric software engineered by Professor Chaomei Chen and his collaborators that facilitates the visualization and analysis of sophisticated citation networks. In this study, the following analyses were performed utilizing CiteSpace (version 6.2.R4 advanced) software, developed by Dr. Chaomei Chen at Drexel University, Philadelphia, PA, USA: mainly the contribution and centrality of keywords and references. R language's bibliometrix package is a powerful and flexible tool that helps researchers perform intensive literature data analysis. In this study, Bibliometrix (R-Studio's R-Tool, version 4.3.2),an R package developed by Massimo Aria and Corrado Cuccurullo at the University of Rome Tor Vergata, Italy, was used to carry out the following analytical tasks: keywords, references, and collaborative network analysis. In addition, Microsoft Excel (version 2021), developed by Microsoft Corporation (Redmond, WA, USA), was used for data analysis of trends in annual publications.

The common bibliometric terms and the criteria for establishing them were as follows: (1) keywords for analysis were extracted from title, abstract, author's keyword, and keyword plus; (2) Node centrality evaluates a node's capacity to function as a connector between other nodes, underscoring its critical role in facilitating information exchange or knowledge transfer. In this analysis, CiteSpace was utilized to calculate node centrality, applying a default threshold of 1; (3) The H-index serves as a composite metric for evaluating the scholarly output and influence of authors or journals. In this analysis, authors' H-index values were calculated using Bibliometrix, while publication counts across countries/regions, authors, journals, and keywords were quantified by VOSviewer; (4) the term “co-cited” describes the occurrence of two or more publications being jointly referenced by subsequent publications, whereas co-citation refers to the observed phenomenon or the bibliometric technique for analyzing these co-occurrence patterns; (5) Citation burst denotes a significant increase in the citation frequency of a publication over a defined time interval. The default threshold in CiteSpace's Burstness function was maintained at default values, with *γ* set to 1. Finally, the top 20 keywords out of 134 and the top 10 references out of 583 were selected for analysis; (6) A dual-map overlay of journals was created through CiteSpace's overlay maps feature, leveraging the journal citation reports (JCR) dual map overlay (v.2.2) platform. The visualization integrated a JCR base map comprising 10,330 journals, courtesy of Loet Leydesdorff.

## 3 Results

### 3.1 Analysis of trends in annual publications

**Figure 2:**
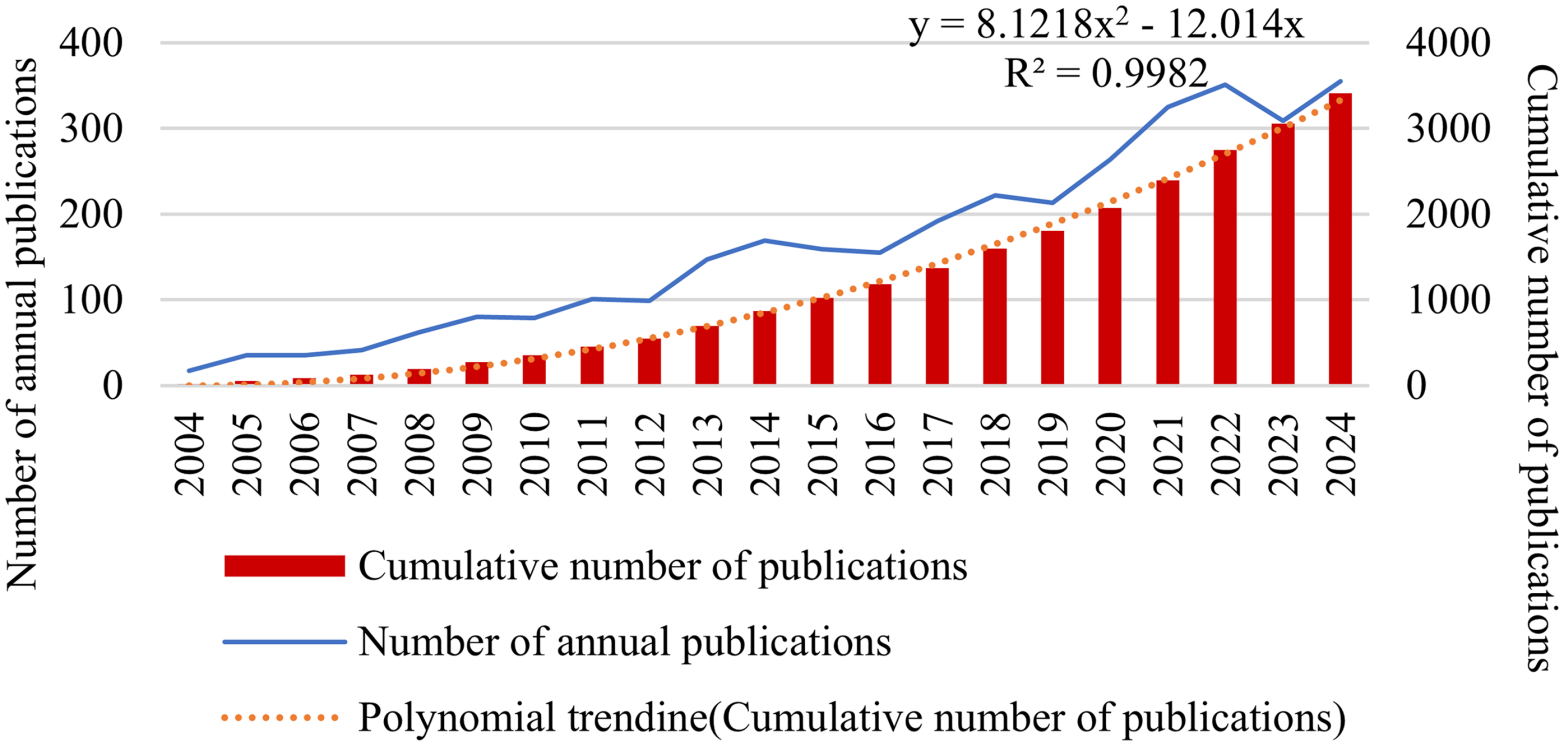
Trend graph of the number of annual publications on MASLD in children (retrieval time span: 01/01/2004–31/12/2024).

The systematic literature search initially retrieved 4,307 records after applying temporal constraints and executing the search query. Limiting the results to English-language publications reduced the count to 4,255. Further refinement by restricting the document type to “article” or “review article” yielded 3,482 publications. Finally, after excluding other document types, such as proceeding papers, a final total of 3,409 relevant publications on MASLD in children remained, including 2,697 articles and 712 reviews. These articles were derived from 562 countries or regions and published in 967 journals. As indicated in Figure 2, a number of annual publications showed a general upward trend between 2004 and 2024, while a slight reduction in 2023, but the number apparently rebounded to the peak in 2024. Particularly from 2012 to 2014 and 2020 to 2022, the increase was relatively significant. The increase from 2016 to 2020 was more moderate, despite a slight decline in publications from 2014 to 2016 and a marginal decrease in 2019. According to the polynomial trendline, the cumulative volume of publications in pediatric MASLD maintained a high growth tendency.

### 3.2 Analysis of the distribution of major countries/regions and institutions

**Figure 3:**
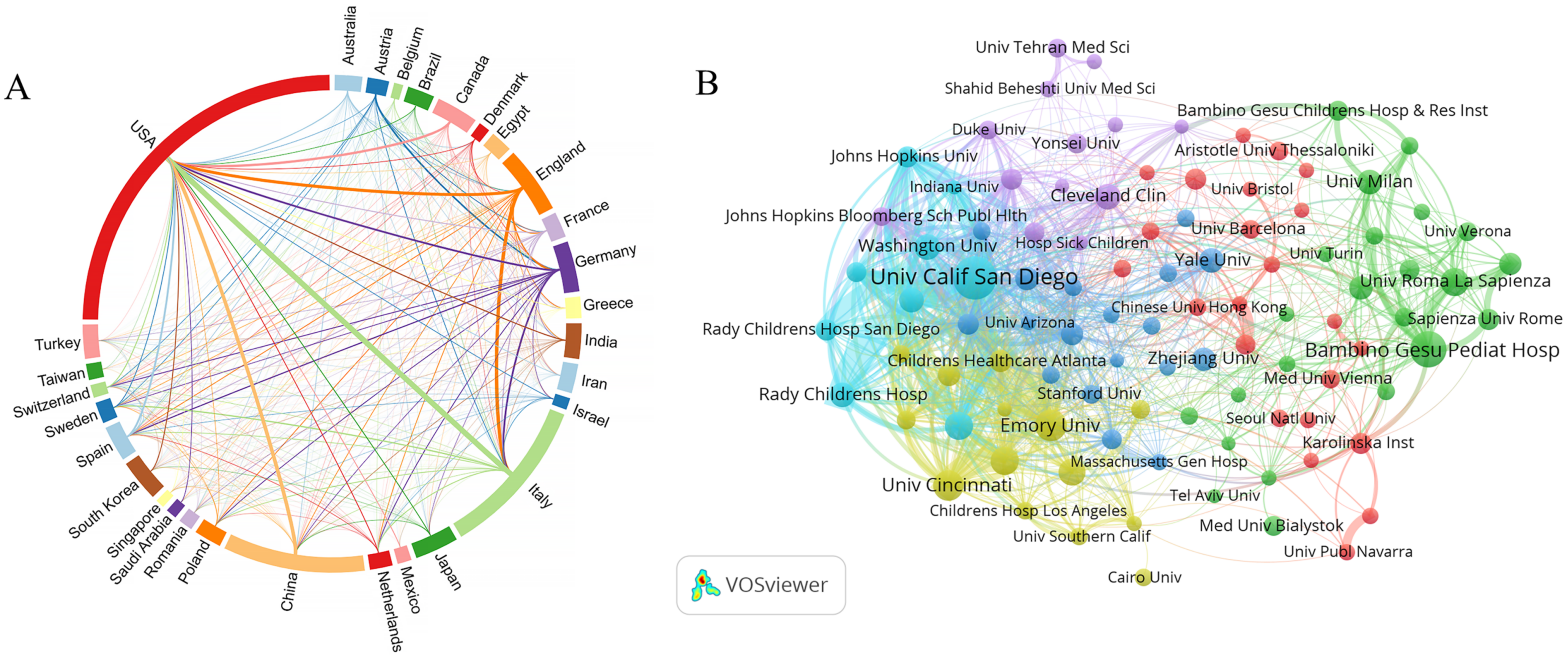
Analysis of countries/regions and institutions. **(A)** National/regional collaborative network. Each label represents a country or region, the length of the arc corresponds to the volume of publications, and the width of the connecting lines indicates the strength or frequency of collaboration. **(B)** Institutional collaborative network in VOSviewer. Each node represents an institution, the size of the nodes represents the number of publications.

The field of pediatric MASLD research covers 562 countries or regions. To identify the countries or regions with the most prominent contributions to the field, VOSviewer was used to analyze the top 30 countries and regions, which is illustrated in Figure 3A; among them, the United States top globally in publications, Italy emerges in second, and China follows in third, and the most vital connections are between United States and Italy, as well as United States and China, closely followed by United States and England, there are also considerable cooperation between Italy and England. In bibliometrics, centrality is a measure of the perceived weight of a node in a network, and according to the results of CiteSpace analysis, the quantity of publications and centrality are factors considered to ascertain the most significant 5 countries or regions, respectively, and the top 5 countries or regions are listed in Table 1. The United States led in terms of the volume of publications (*n* = 1,145 publications), meanwhile it ranked first in terms of centrality with a share of 0.29.

**Table 1:** Top 5 countries/regions in terms of number of publications and centrality in the field of pediatric MASLD research from 2004 to 2024.

Rank	Countries/regions	Publications	Countries	Centrality
1	USA	1145	USA	0.29
2	Italy	485	England	0.26
3	China	429	Canada	0.18
4	England	203	Italy	0.15
5	Germany	153	Spain	0.11

The analysis of research institutions seeks to capture the worldwide dissemination of research associated with the pediatric MASLD field as a reference for researchers seeking collaborations. The top 5 institutions are ranked according to the number of publications as well as centrality, which are shown in Table 2, and the University of California System had both the largest volume of publications (*n* = 214), as well as the highest centrality with a centrality share of 0.14. In addition, the Istituto di Ricovero e Cura a Carattere Scientifico (IRCCS) ranked second in publications (*n* = 202), and it tied with University of California System for first place in terms of centrality. As illustrated in Figure 3B, the VOSviewer analysis reveals that institutional collaborations can be broadly categorized into six closely related blocks.

**Table 2:** Top 5 institutions in terms of number of publications and centrality in the field of pediatric MASLD research from 2004 to 2024.

Rank	Institution	Publications	Institution	Centrality
1	University of California System(USA)	214	University of California System (USA)	0.14
2	IRCCS Bambino Gesu (Italy)	202	IRCCS Bambino Gesu (Italy)	0.14
3	University of California San Diego (USA)	160	University of California San Diego (USA)	0.10
4	University System of Ohio (USA)	95	Washington University (WUSTL) (USA)	0.10
5	Sapienza University of Rome (Italy)	93	University of London (England)	0.10

### 3.3 Analysis of active authors and co-cited authors

The active authors and co-cited authors analysis assist in identifying critical figures in the field of pediatric MASLD. Co-cited authors are commonly scholars with high influence in the same field, and their research achievements are recognized as instrumental to the development and promotion of the field. The first 5 authors and co-cited authors are shown in Table 3 for their respective publication volumes and institutions. Among them, Valerio Nobili (Bambino Gesu Pediat Hosp, Italy) had the maximum amount of publications with a total of 169 papers, and he was the pioneer in the field of pediatric MASLD, Anna Alisi (Children's Hosp & Res Inst Bambino Gesu, Italy) ranked second with a volume of 121 publications and Jeffrey Schwimmer (Univ Calif San Diego, USA) ranked third with 74 publications. By analyzing the co-cited authors, it could be concluded that Jeffrey Schwimmer (*n* = 2,004), a scholar from the USA, had the highest citation level and had made a major contribution to the field of pediatric MASLD, with Valerio Nobili (*n* = 1,855) in the second rank, and Zobair Younossi (*n* = 860) in the third rank. It was noteworthy that Valerio Nobili ranked first for the quantity of publications in this area and ranked second in terms of citation level.

**Table 3:** Top 5 authors and co-cited authors in the field of pediatric MASLD research from 2004 to 2024.

Rank	Author	Publications	H-index	Institution	Co-cited author	Citations	Institution
1	Valerio Nobili	169	62	Bambino Gesu Pediat Hosp (Italy)	Jeffrey Schwimmer	2,004	Univ Calif San Diego (USA)
2	Anna Alisi	121	48	Childrens Hosp & Res Inst Bambino Gesu (Italy)	Valerio Nobili	1,855	Bambino Gesu Pediat Hosp (Italy)
3	Jeffrey Schwimmer	74	44	Univ Calif San Diego (USA)	Zobair Younossi	860	Inova Fairfax Hosp (USA)
4	Stavra Xanthakos	59	20	Univ Cincinnati (USA)	Elizabeth Brunt	672	Washington Univ (USA)
5	Miriam Vos	54	27	Columbia Univ (USA)	David Kleiner	639	National Cancer Institute (USA)

**Figure 4:**
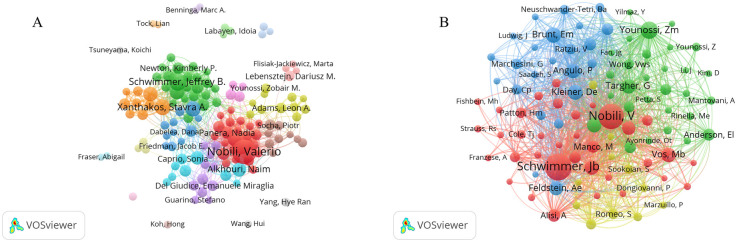
Analysis of active authors and co-cited authors. **(A)** Collaborative network of authors in VOSviewer. Each node represents an author, the size of the nodes represents the quantity of publications, the thickness of the lines reflects the closeness of the cooperation, the colors are applied to distinguish different cooperation clusters. **(B)** Collaborative network of co-cited authors in VOSviewer. Each node represents a co-cited author. Connecting lines indicate co-citation relationships, with thicker lines showing higher co-citation frequency. Authors are clustered by citation patterns, revealing shared research themes or methodologies within each cluster.

The collaborations of the authors of literature on pediatric MASLD are displayed in VOSviewer (Figure 4A), which can be roughly divided into 9 clusters centered on Valerio Nobili and Jeffrey Schwimmer. The network map of co-cited authors indicated a high degree of homogeneity in the study focus of authors in the literature on pediatric MASLD (Figure 4B), which could be divided into 4 main clusters.

### 3.4 Analysis of journals distribution and co-cited journals

A bibliometric analysis of journals and co-cited journals in the domain of pediatric MASLD allowed for the identification of highly productive and influential journals. The list of the top 5 journals with the highest number of publications is summarized in Table 4. *Journal of Pediatric Gastroenterology and Nutrition* (IF 2.4, Q3/Q1) was the most productive journal in terms of quantity, ranking first with a total of 125 publications. *Hepatology* (IF 13, Q1) and *World Journal of Gastroenterology* (IF 4.3, Q1) were the second and third journals with 77 and 62 publications. The 5 most co-cited journals with the highest frequency of citations are displayed in Table 5, and the top 3 journals were *Hepatology* (IF 13, Q1), *Journal of Hepatology* (IF 26.8, Q1), and *Gastroenterology* (IF 26.3, Q1). As evidenced, a multitude of studies on pediatric MASLD have been conducted based on the outcomes of research published in these journals.

**Table 4:** Top 5 journals in terms of total number of publications in the field of pediatric MASLD research from 2004 to 2024.

Rank	Journal	Publications	IF (JCR 2023)	JCR quartile
1	*Journal of Pediatric Gastroenterology and Nutrition*	125	2.4	Q3/Q1
2	*Hepatology*	77	13	Q1
3	*World Journal of Gastroenterology*	62	4.3	Q1
4	*Nutrients*	62	4.8	Q1
5	*Plos One*	61	2.9	Q1

IF, impact factor; JCR, journal citation reports.

**Table 5:** Top 5 co-cited journals in terms of total number of citation frequency in the field of pediatric MASLD research from 2004 to 2024.

Rank	Journal	Citation	IF (JCR 2023)	JCR quartile
1	*Hepatology*	12,169	13	Q1
2	*Journal of Hepatology*	5,569	26.8	Q1
3	*Gastroenterology*	5,185	26.3	Q1
4	*Journal of Pediatric Gastroenterology and Nutrition*	3,200	2.4	Q3/Q1
5	*PLoS One*	2,800	2.9	Q1

IF, impact factor; JCR, journal citation reports.

**Figure 5:**
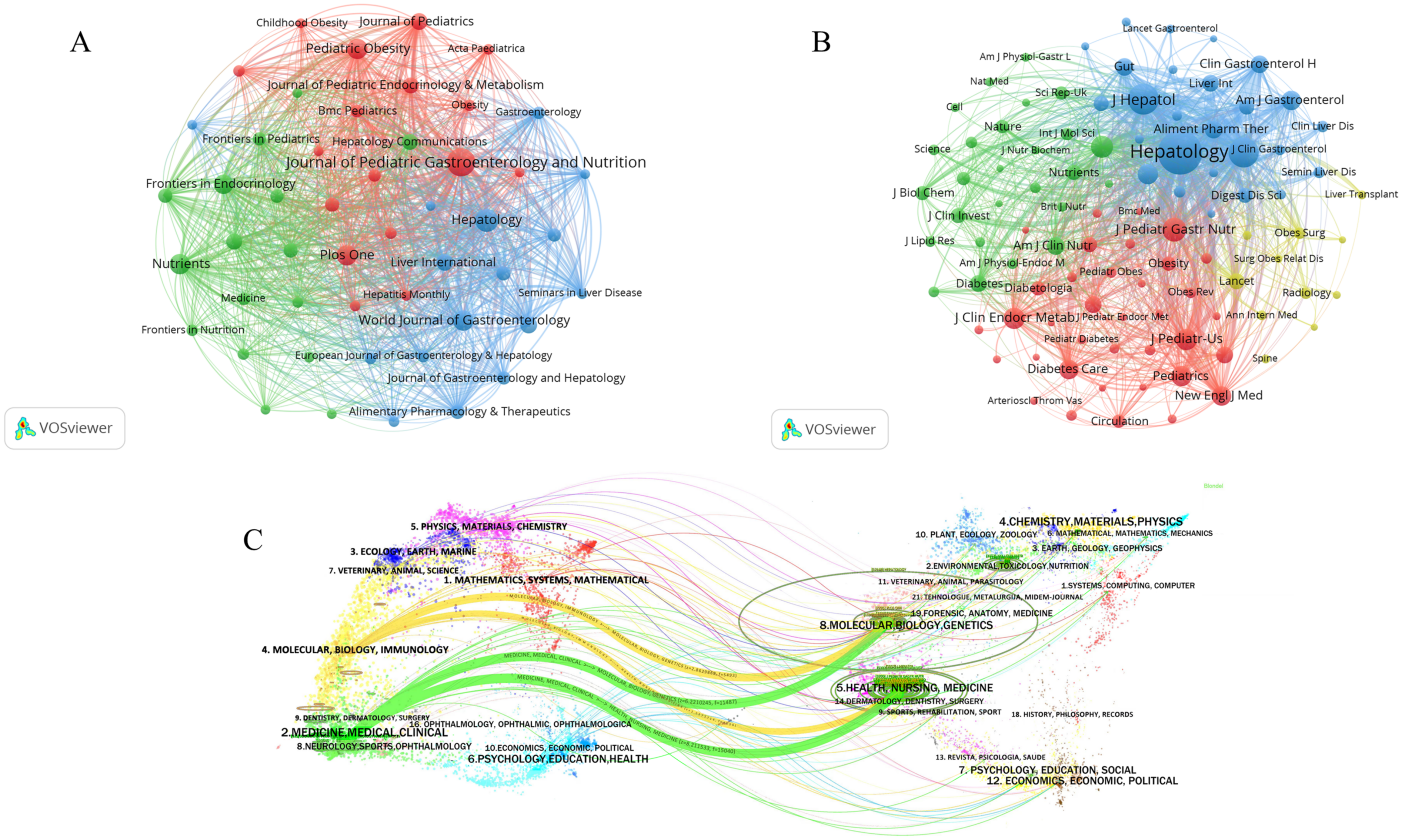
Analysis of journals and co-cited journals. **(A)** Collaborative network of journals in VOSviewer. Each node represents a journal, the size of the nodes represents the number of publications, the connecting lines between the nodes indicate the cooperation, and the colors are applied to distinguish different cooperation clusters. **(B)** Collaborative network of co-cited journals in VOSviewer. Each node represents a co-cited journal. The connecting lines quantify the relationship strength between nodes, with thicker lines indicating higher co-citation frequencies. Journal clusters are color-coded, and journals within the same cluster exhibit similar research themes or disciplinary domains. **(C)** The dual-map overlay of journals. The left panel illustrates research frontiers formed by citing journals, while the right panel represents the intellectual base composed of cited journals. Each node represents a journal, with distinct colors indicating different journal categories. Each category is labeled with its cluster theme. Connecting lines depict citation relationships between citing and cited journals, where line colors denote distinct citation paths. Key paths are annotated with *z*-score and f score (frequency of citations), where *z*-score is a standardized value of f score. Thicker lines indicate higher *z*-scores. On the left side, the horizontal axis of the ellipse represents the publication volume, while on the right side, it reflects the citation count. Thus, on the left side, a longer horizontal axis indicates a higher number of papers published in the corresponding journal. A longer vertical axis signifies a greater number of authors.

The results of journals and co-cited journals in the domain of pediatric MASLD were visualized and analyzed by VOSviewer. Figure 5A shows the distribution of journals that study pediatric MASLD and the relationship between them, which could be mainly divided into 3 clusters indicated by various colors. *Journal of Pediatric Gastroenterology and Nutrition* and *Hepatology*, *Nutrients* emerged as the centers of the various clusters, and they were also tightly connected to each other. Figure 5B shows the distribution of cited journals and the association, which can be categorized roughly into 4 clusters, with *Hepatology* being the most prominent node and closely related to *Gastroenterology* and *Journal of Hepatology.* Via CiteSpace analysis, Figure 5C shows the dual-map overlay of journals on the pediatric MASLD field. On the left of the figure was a collection of citing journals, representing the current knowledge front, and on the right was a collection of cited journals, representing the knowledge foundation of the research area. The connecting lines between them reflected the macro structural development pattern of the field, indicating that research in pediatric MASLD exhibits a macroscopic development model characterized by the intersection of disciplinary themes.

### 3.5 Keywords co-occurrence and burst

**Figure 6:**
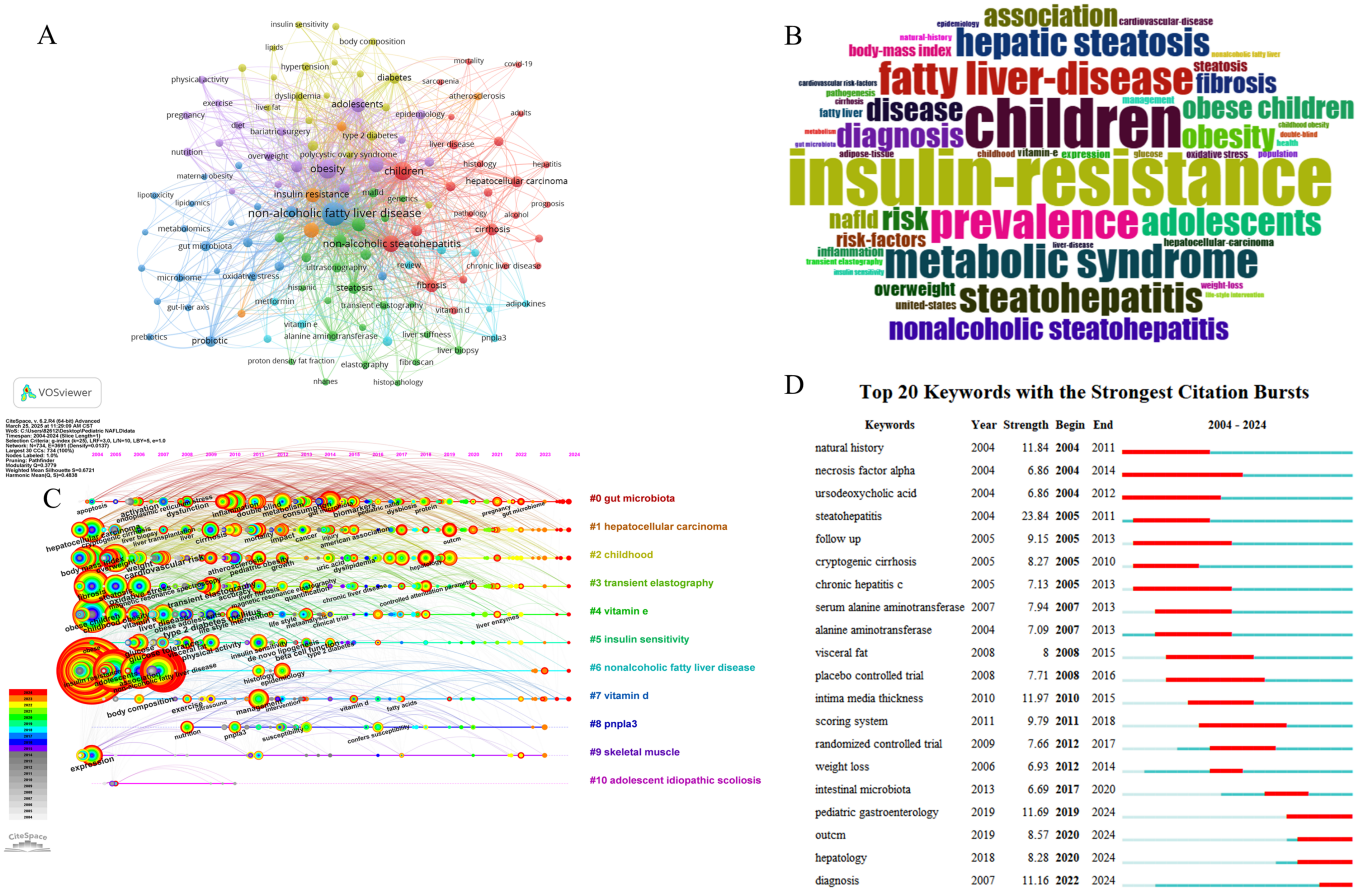
Analysis of keywords. **(A)** Collaborative network of keywords in VOSviewer. Each node represents a keyword, with its size indicating frequency. Connecting lines indicate keyword co-occurrence in publications, thicker lines mean higher co-occurrence frequency. Color-coded clusters group related keywords by research theme. **(B)** Keywords cloud map. Larger font size indicates higher frequency of occurrence. **(C)** Timeline view of keywords. The diagram uses circular nodes to denote keywords, where node size corresponds to keyword frequency. A color spectrum from dark to light indicates the chronological order, and lines between nodes signify keyword associations. **(D)** Top 20 keywords with the strongest citation bursts. The blue line indicates the time of emergence, and the red line indicates that the literature was cited with high frequency during this period. The keyword “outcm” is the abbreviation of “outcome”. “Strength” quantifies the burst intensity of keywords within a defined temporal framework, representing the amplitude of abrupt research interest growth.

Keyword analysis is an essential component of bibliometrics, contributing to our knowledge of the frontiers and hotspots of the research field. Table 6 displays the top 20 keywords ranked by frequency of occurrence, with “non-alcoholic fatty liver disease” (*n* = 1,379) having the highest frequency of occurrence, followed by “obesity” (*n* = 622) and “children” (*n* = 493). Visual analysis of the keywords by VOSviewer resulted in a co-occurrence network map, as shown in Figure 6A, with approximately seven clusters represented by distinct colors. In particular, the red clusters were primarily associated with pathological alterations, such as “non-alcoholic steatohepatitis”, and “cirrhosis”. The purple clusters were associated with obesity, such as “adolescent”, “overweight”, and “body mass index”. The green clusters were mainly related to clinical tests and diagnostics, such as “ultrasonography”, “alanine aminotransferase”, “liver biopsy”. The keyword word cloud generated according to the R-studio software is shown in Figure 6B. A word cloud is a visualization approach used to show the frequency of keywords in textual data, which is very intuitive and straightforward, and usually, the keywords are visualized in the same size as the frequency of their occurrence; hence, “insulin-resistance”, “children”, “metabolic syndrome”, “steatohepatitis”, “prevalence”, for example, are popular research topics in the area.

**Table 6:** Top 20 keywords in terms of frequency of occurrences in the field of pediatric MASLD research from 2004 to 2024.

Rank	Keyword	Occurrences	Rank	Keyword	Occurrences
1	Non-alcoholic fatty liver disease	1,379	11	Fibrosis	124
2	Obesity	622	12	Steatosis	111
3	Children	493	13	Cirrhosis	105
4	Non-alcoholic steatohepatitis	380	14	Liver	85
5	Metabolic syndrome	224	15	Inflammation	82
6	Fatty liver	224	16	Childhood obesity	79
7	Insulin resistance	218	17	Hepatocellular Carinoma	78
8	Adolescents	200	18	Diabetes	74
9	Hepatic steatosis	157	19	Probiotic	70
10	Pediatric	139	20	Alanine aminotransferase	60

The keywords were visualized and analyzed by CiteSpace, and a keyword timeline graph (Figure 6C) was derived. The timeline graph displayed the keywords that appeared most frequently in the various clusters throughout a specific period, indicating that the keywords in the field of pediatric MASLD research could be classified into 11 clusters, of which the earliest and largest cluster was #0 (gut microbiota), the keyword “apoptosis” appeared previously, and “gut microbiome” was a relatively updated keyword from 2022 onwards. Another critical clustering was #1 (hepatocellular carcinoma), an axis where the previous keyword was “hepatocellular carcinoma”, but “outcome” was at the forefront. Eight clusters are still in progress, including #0 (gut microbiota), #1 (hepatocellular carcinoma), #2 (childhood), #3 (transient elastography), #4 (vitamin e), #5 (insulin sensitivity), #6 (nonalcoholic fatty liver disease), #7 (vitamin d). Figure 6D shows the Top 20 keywords with the strongest citation bursts. It was worth noting that the keywords that were more popular in the last few years were “pediatric gastroenterology”, “outcome”, “hepatology” and “diagnosis”. In contrast, the keyword with the highest burst strength was “steatohepatitis” at 23.84. The second was “intima media thickness”, with a burst strength of 11.97.

### 3.6 Analysis of co-cited references and references with citation bursts

Co-cited references analysis, a core feature of CiteSpace, identifies pivotal research clusters or paradigms within a field by quantifying the co-citation frequency between publications. Highly co-cited references typically represent foundational studies in the discipline. The top 10 articles in the domain of pediatric MASLD in terms of co-citation frequency are listed in Table 7, where the top co-cited publication was published in the *Journal of Pediatric Gastroenterology and Nutrition* in 2017 by Miriam Vos, et al. This publication was a clinical practice guideline for the identification and management of pediatric MASLD. The publication detailed the diagnostic criteria, etiology, assessment methods, and treatment strategies for pediatric MASLD, and played an essential role in helping physicians and clinicians identify and manage pediatric MASLD better (20). The second-ranked publication was also a clinical guideline that provided clinicians with a reliable basis for diagnosing, treating, and preventing MASLD (21). The third-ranked publication was a meta-analysis that analyzed the prevalence of MASLD in children or adolescents between the ages of 1 and 19 years and the correlation with factors such as body mass index (BMI) (22). Among the other seven publications, three studies investigated the prevalence of MASLD in children or adolescents, along with related outcomes and trends (4, 23, 24); two articles proposed recommendations for renaming NAFLD to MAFLD (26, 27), and the remaining two explored the diagnostic and therapeutic aspects of MASLD in this age group.

**Table 7:** Top 10 co-cited references in the field of pediatric MASLD research from 2004 to 2024.

Rank	Article title	Source title	First author	Year	Co-citation count
1	NASPGHAN clinical practice guideline for the diagnosis and treatment of nonalcoholic fatty liver disease in children: recommendations from the expert committee on NAFLD (ECON) and the North American Society of Pediatric Gastroenterology, Hepatology and Nutrition (NASPGHAN) (20)	*Journal of Pediatric Gastroenterology and Nutrition*	Miriam Vos	2017	259
2	The diagnosis and management of nonalcoholic fatty liver disease: Practice guidance from the American Association for the Study of Liver Diseases (21)	*Hepatology*	Naga Chalasani	2018	160
3	The prevalence of non-alcoholic fatty liver disease in children and adolescents: a systematic review and meta-analysis (22)	*PLoS One*	Emma Anderson	2015	127
4	Global epidemiology of nonalcoholic fatty liver disease-meta-analytic assessment of prevalence, incidence, and outcomes (23)	*Hepatology*	Zobair Younossi	2016	120
5	Global burden of NAFLD and NASH: trends, predictions, risk factors and prevention (24)	*Nature Reviews Gastroenterology & Hepatology*	Zobair Younossi	2018	119
6	Prevalence of fatty liver in children and adolescents (4)	*Pediatrics*	Jeffrey Schwimmer	2006	109
7	NAFLD in children: new genes, new diagnostic modalities and new drugs (25)	*Nature Reviews Gastroenterology & Hepatology*	Valerio Nobili	2019	105
8	A new definition for metabolic dysfunction-associated fatty liver disease: an international expert consensus statement (26)	*Journal of Hepatology*	Mohammed Eslam	2020	96
9	MAFLD: a consensus-driven proposed nomenclature for metabolic associated fatty liver disease (27)	*Gastroenterology*	Mohammed Eslam	2020	95
10	Diagnosis of nonalcoholic fatty liver disease in children and adolescents: position paper of the Espghan hepatology committee (28)	*Journal of Pediatric Gastroenterology and Nutrition*	Pietro Vajro	2012	92

**Figure 7:**
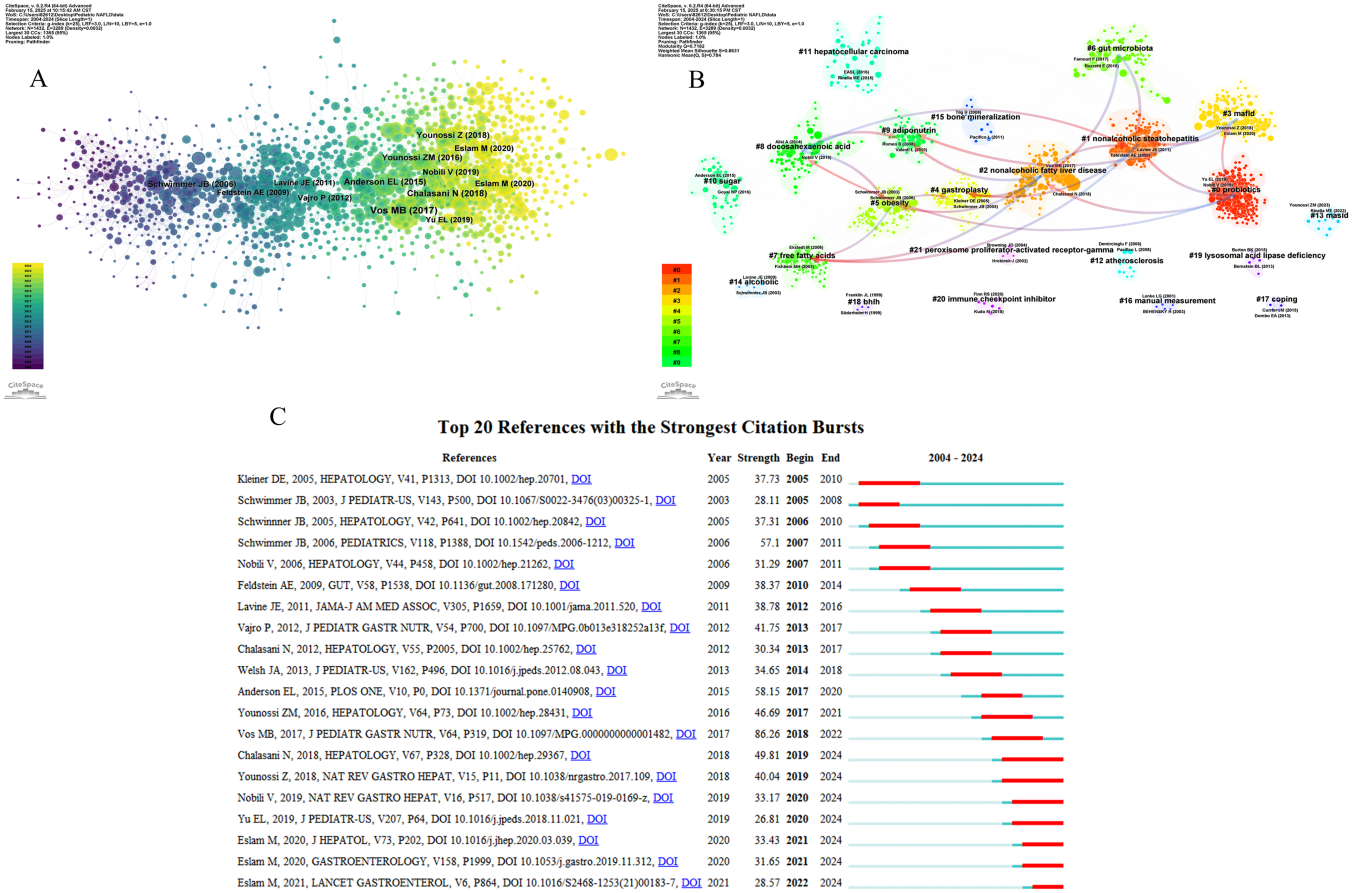
Analysis of co-cited references. **(A)** Collaborative network of references in CiteSpace. Each node represents a reference, the size of the node represents the number of citations, the line between the nodes indicates the co-citation relationship between the two documents, and the color of the node indicates the year change. **(B)** References clustering map. The color blocks in the figure represent a cluster, the cluster number is inversely proportional to the amount of literature, the large label is the name of the cluster, the small label is the main representative literature, and the red line with arrows reflects the origin of each cluster. **(C)** Top 20 references with the strongest citation bursts. On the left side of the figure are the bursts of literature. “ Strength” is the key indicator of burst strength, and the red line on the right side represents the duration of the bursts.

As illustrated in Figure 7A, CiteSpace software was utilized to analyze and generate a network map, where every single node referred to a publication, the magnitude of a node reflected its citation frequency, the connectivity between the nodes showed the existence of a co-occurrence relationship between them, and the variation in node color represented the time of the publication's appearance. As seen in Figure 7B, the publications can be classified into 22 clusters depending on the degree of association between them, and the lines in red with arrows reflect the previous origins of the clusters. The most important and earliest cluster to emerge was #0 (probiotics), which developed 4 clusters, including #1 (nonalcoholic steatohepatitis), #2 (nonalcoholic fatty liver disease), #3 (MAFLD), #8 (docosahexaenoic acid), the clustering that immediately follows were #1 (nonalcoholic steatohepatitis), #2 (nonalcoholic fatty liver disease), the more contemporary research clustering were #19 (lysosomal acid lipase deficiency), #20 (immune checkpoint inhibitor), #21 (peroxisome proliferator-activated receptor-gamma). Figure 7C shows the topics in the top 20 references with the strongest citation bursts. The analysis revealed that there were 7 publications that were still in the thermal phase, and the most significant and longest-lasting citation burst after 2019 was a practice guidance authored by Naga Chalasani, et al. and published in *Hepatology*, with a burst strength of 49.81. Immediately following this was an article published in *Nature Reviews Gastroenterology & Hepatology*, with a burst strength of 40.04. The most recent article was authored by Mohammed Eslam and published in 2021.

### 3.7 Multivariate relational network analysis

**Figure 8:**
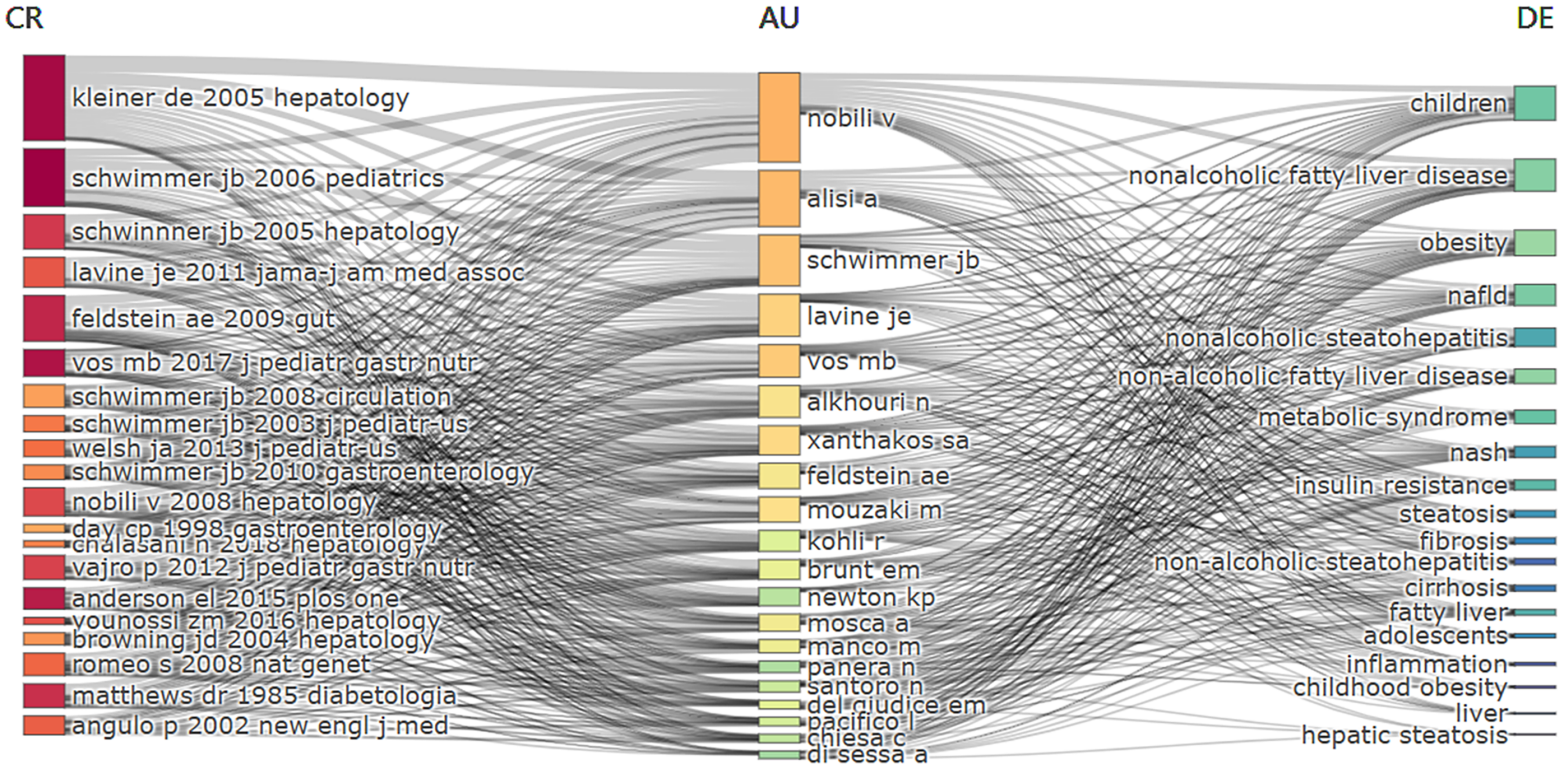
Three-field plot of pediatric MASLD related study. The labels “CR”, “AU” and “DE” represent “cited references”, “author” and “keywords”, respectively. The entries in each field are sorted by frequency, the connecting lines between different fields indicate the associations between the entries, and the width of the lines reflects the strength of the connections. Different color shades used in the bars within a particular field represent changes in the strength of information, such as frequency, intensity, importance, time dimension, or research hotspots.

The link between the three variables was shown by the three-field plot analysis, which includes references, authors, and keywords. The intensity of the associations was indicated by the width of the connecting lines, while the height of the rectangles represents the amount of publications. As illustrated in Figure 8, Valerio Nobili, Anna Alisi, and Jeffrey Schwimmer ranked among the top three based on the width of their entries, which indicates a higher frequency, while these authors also referenced highly cited references. Valerio Nobili extensively cited several of the references listed on the left in his own articles, with the most frequently cited being David Kleiner's article published in 2005. Meanwhile, Valerio Nobili's study involved the keywords “children” and “nonalcoholic fatty liver disease” with high frequency, as well as “obesity”, “insulin resistance”, “metabolic syndrome” and “fibrosis”.

## 4 Discussion

MASLD has become the most prevalent liver lesion in children and adolescents, and this study strives to summarize the frontiers and hotspots in the domain of pediatric MASLD for the assistance of scholars. CiteSpace (6.2.R4) Advanced, VOSviewer (1.6.20), and bibliometrix were utilized to conduct bibliometrics and visualization studies on 3,409 publications retrieved from Web of Science in the field of pediatric MASLD between 2004 and 2024, revealing key authors, spatial and temporal distribution, core keywords, critical citations, and other information in order to discover trends and hotspots in this domain.

### 4.1 Summary of publications

#### 4.1.1 Analysis of the volume of publications in the field of pediatric NAFLD study

Between 2004 and 2024, the number of annual publications showed a steady upward trend, indicating that pediatric MASLD is increasingly emphasized by clinicians and researchers. After 2020, global infectious diseases have been controlled to a certain extent, and pediatric non-infectious diseases have been gradually highlighted. Meanwhile, dietary excess and reduced outdoor activities have probably contributed to the rise in the prevalence of childhood obesity, thus leading to the increased diagnosis rate of pediatric MASLD, which may be the reason for the accelerated growth of publications from 2020 to 2022.

#### 4.1.2 Analysis of the contribution of countries/regions, institutions, authors, and journals of pediatric MASLD

Among the 562 countries or regions studying pediatric MASLD, the top 3 in the volume of publications are the USA, Italy, and China, and USA is the most centrally located, which plays a leading role in pediatric MASLD study. There are also strong links and collaborations among them to promote the global research progress in pediatric MASLD study. Regarding to the quantity of publications, 3 of the top 5 organizations are from the United States, and 2 are from Italy; in terms of centrality, 3 of the top 5 organizations are from the United States, and 1 is from Italy. Moreover, there is extensive cooperation and exchange among the institutions.

As presented in Table 3; Figure 4, Valerio Nobili of Italy has the highest amount of publications and is also the second most highly co-cited author, which may be related to his highly co-cited article published in *Nature Reviews Gastroenterology & Hepatology* in 2019. This highly co-cited article contributed significantly to the development of pediatric MASLD research.

As demonstrated by Tables 4, 5; Figure 5, the *Journal of Pediatric Gastroenterology and Nutrition* not only has the most publications of pediatric MASLD studies but is also the number 4 of the 5 journals in terms of number of co-citation frequency. *Hepatology* had the highest co-citation frequency and ranked second in terms of publication volume, indicating that these journals have contributed a significant volume of pivotal articles on pediatric MASLD research and exhibit a high index of influence.

### 4.2 Hotspots and frontiers

Keyword and citation analysis is the core content and advantage of bibliometric research, which can help fellows to understand the dynamics and focus of the field quickly and to catch the core literature and emerging research directions. According to Table 6; Figure 6, the keyword analysis indicates that in the field of pediatric MASLD study, the pathological lesions of the liver, the connection with obesity, the complications, and comorbidities, the diagnosis and treatment of MASLD are the focus of the researchers. The gut microbiota has emerged as a prominent and enduring research focus within this domain, evidenced by its sustained high citation frequency over an extended period. Still, the newest hotspots in the last few years mainly focus on the diagnosis, risk, and outcomes of pediatric MASLD.

#### 4.2.1 Pathologic, natural history studies of pediatric MASLD

According to estimations, 38.0% of obese children and 9.6% of children between the ages of 2 and 19 in the United States have fatty livers (4), and studies have shown that children have a potentially riskier prognosis for fatty liver compared to adults (24). Adult liver-related mortality, hepatocellular carcinoma, and cirrhosis are all associated with elevated BMI in childhood or youth (8). The diagnosis of nonalcoholic fatty liver disease (NAFL) is established when there is the presence of more than 5% hepatic steatosis (20). As an exclusionary diagnosis, NAFL is fundamentally driven by metabolic dysfunction, which underlies disease progression. NAFLD is a complex spectrum of progressive clinicopathology of the liver that begins as NAFL, commences with simple steatosis, and can advance to nonalcoholic steatohepatitis (NASH) characterized by liver inflammation and possibly liver fibrosis, followed by cirrhosis and hepatocellular carcinoma (HCC) associated with NAFLD (29). Some discrepancies exist in prevalence, risk propensity, and liver histology in children compared to adults with NAFLD (30). From 2020 onwards, MAFLD has been a hot topic of research, and it is proposed to supersede prior terminology used for the condition known as metabolic dysfunction-associated fatty liver disease (8). However, in 2023, multiple international liver associations proposed at the annual European Liver Congress to name metabolism-associated steatohepatopathy as metabolic dysfunction-associated steatotic liver disease (9).

The pathogenesis of the disease is associated with hepatic fat accumulation and insulin resistance; concomitantly, “second hits” occur, such as inflammatory cytokines, adipokines, mitochondrial dysfunction, and oxidative stress (31). Simultaneously, genetic variants are another critical factor (32); numerous sequence variations, including missense mutations in *PNPLA3*, *TM6SF2*, *GCKR*, and *MTARC1*, have been linked to MASLD (33), and some differences exist in genetic variation between children and adults (34). Fructose metabolism is also a hot topic in current research, with studies showing that fructose intake leads to hepatic insulin resistance (35), gut microbiome, and gut-liver axis dysfunction, *Oscillospira* spp. are relatively uncommon in children with MASLD/MASH, but *Dorea*, *Blautia*, *Prevotella copri*, and *Ruminococcus* spp. are considerably more common, nevertheless, it is challenging to make any conclusive or causal conclusions about the characterization of the gut microbiota in patients with MASLD (36). Simultaneously, it is still unknown how water-electrolyte metabolism functions in children with MASLD (37). The mechanism of development from steatosis to steatohepatitis is poorly defined, and it is uncertain whether MASLD in children and adults are essentially distinct entities of pathology or simply an age-dependent presentation of the same disease (38).

MASLD in adults is strongly associated with complications and outcomes, including overweight/obesity, hyperlipidemia/dyslipidemia, hypertriglyceridemia, hypertension, metabolic syndrome, diabetes, and fibrosis progression (23). These conditions are also present in pediatric patients (39). It has now been established that pediatric MASLD is related to cardiometabolic risk (40), chronic kidney diseases (41), as well as polycystic ovarian syndrome, obstructive sleep apnoea and osteopenia (25). Furthermore, according to Figure 6C, hepatocellular carcinoma, cardiometabolic risk, diabetes risk and low bone mineral density are foci of academic concern, the affiliations between each of these diseases are also a hot topic of current research.

#### 4.2.2 Diagnosis of pediatric MASLD

Given the severe complications and outcomes of MASLD in children, we emphasize the paramount importance of early diagnosis and diagnostic accuracy; however, typical clinical manifestations are most often seen in children aged 10 years and above, and the primary methods for diagnosing MASLD in children nowadays include serum biomarkers and imaging tests. However, liver biopsy is still considered the gold standard for diagnosing the disease (28). In the quest for a diagnostic method that is noninvasive, precise, sensitive, and appropriately priced, the experts have conducted numerous explorations and studies to judge the extent of hepatic steatosis, inflammation, and fibrosis from multiple perspectives, including diagnostic markers for MASLD and liver fibrosis markers.

Among the multitude of serum biomarkers, alanine aminotransferase (ALT) remains a widely applied and inexpensive clinical diagnostic of MASLD in children, but it has lower sensitivity and lacks thresholds for standardized liver enzymes in children; in addition, markers of inflammation, oxidative stress, apoptosis, and fibrosis have been utilized, but studies with large samples are lacking. For example, some findings have revealed a correlation between serum hyaluronic acid (HA) values and the level of liver fibrosis in pediatric MASLD. In addition, proteomic methodologies are emerging areas of research in this field (28). The anti-aging gene Sirtuin 1 may be more sensitive to inactivation in children with the early changes in the liver and the induction of MASLD. Sirtuin 1 activation vs. Sirtuin 1 inhibition may need to be assessed with relevance to the prevention of MASLD (42). Nutritional interventions are critical for the prevention of MASLD in both developed and developing world (43), bacterial lipopolysaccharides (LPS) play a significant role in the pathogenesis of MASLD through the gut-liver axis, and plasma LPS levels may assist with the use of serum biomarkers (44).

Imaging tests are helpful for quantifying hepatic steatosis (computed tomography, magnetic resonance imaging, or magnetic resonance spectroscopy) and fibrosis (transient elastography) (25), all of which have their respective advantages and disadvantages. Ultrasonography is most widely used in clinical practice, but it has limitations in quantifying steatosis. In the search for new noninvasive imaging tests, recent studies have suggested that transient elastography (45), magnetic resonance elastography (46), and acoustic radiation force impulse imaging (47) as a relatively innovative diagnostic technique currently may replace liver biopsy for determining the degree of hepatic fibrosis. However, the accuracy of these tests in the pediatric population and the feasibility of their use require further study and attention.

Liver biopsy remains untouched and is still the “gold standard” for pediatric MASLD, which can help define the type of liver injury as well as grading and staging disease severity, although not recommended as a general screening test, and it have inherent limitations in the biopsy procedure and risks to children and adolescents (48). Due to the histopathologic specificity of MASLD in children, efforts will be required to formulate positive diagnostic criteria for subphenotypic staging, feasibility, and compatibility with children (20).

#### 4.2.3 Treatment for pediatric MASLD

The prevention and treatment of pediatric MASLD focuses on lifestyle intervention (49) by modifying the diet and moderate physical activity to maintain an average body mass index to decrease liver enzymes and improve liver histopathology (50). However, due to the specificity of the pediatric population, the weight management program has been implemented unsatisfactorily in the clinic (51). Meanwhile, the routine use of medications for the therapy of pediatric MASLD is not recommended, and no pharmacologic agents are presently authorized for the therapy of pediatric MASLD due to the lack of evidence-based medical proof (20). Sarcopenia has emerged as a risk factor for MASLD and pediatric MASLD is expected to present with co-morbid sarcopenia (52). In non-obese children, low skeletal muscle mass may be an important risk factor for MASLD, so building muscle through weight training as an adjunct to other forms of exercise may be effective in preventing and improving MASLD (53). Medications and supplementation that are frequently used clinically include the extensively studied anti-oxidant vitamin E (54), insulin sensitizer metformin (55), as well as omega-3 fatty acids (56), probiotics or ursodeoxycholic acid, lack multicenter, high-grade, long-term clinical trial studies. Additionally, the utilization of bariatric surgery for the therapy of pediatric MASLD also requires careful consideration (57). In addition, natural products with anti-inflammatory, antioxidant and immunomodulatory effects are also future directions for research on strategies to manage MASLD in children (58).

Currently, emerging research directions for study are being explored in the management and prevention of pediatric MASLD, including the development of interventions related to the gut microbiome, such as fecal microbiota transplantation, the delivery of probiotics, prebiotics, synbiotics, antibiotics, and bacteriophages (36), as well as the addition of omega-3 long-chain polyunsaturated fatty acids as supplements, docosahexaenoic acid, for instance (59). Some new medications that have been studied in adult MASLD patients are expected to be used in pediatrics in the future, such as obeticholic acid, selonsertib, cenicriviroc, aramchol, losartan (25), and elafibranor as a peroxisome proliferator-activated receptor β/δ agonist (60), aiming to improve lipid metabolism and liver fibrosis. However, these preventive and therapeutic measures are still under investigation, the impact on safety indicators such as growth and development also needs to be taken into account, and more convincing evidence to support their widespread clinical application is lacking temporarily (25).

### 4.3 Limitations

To the greatest extent of our understanding, this is the first study that explores academic developments and hot spots in pediatric MASLD from 2004 to 2024, utilizing a bibliometric method and visualization study. However, this paper has some limitations due to various factors. Initially, only one database was analyzed in this study, although WoSCC has precedent for use in bibliometric studies. Secondly, due to challenges in data standardization and language processing, only English literature was selected for analysis, which may have affected the results. Finally, our findings are time-sensitive due to the dynamic updating of the WoSCC database and the lag in the publication of literature. In addition, the diversity of the names of the organizations may cause bias in the research data. Whereas these inevitable flaws and deficiencies are visible, this study conducted the most insightful exploration and analysis of the current state of research in pediatric MASLD.

## 5 Conclusion

With the increasing annual growth in the prevalence of pediatric obesity, MASLD has emerged as a significant and dramatic global public health issue, and the overall trend of the volume of publications of pediatric MASLD has shown a significant yearly increase in the last few years, demonstrating that this domain is receiving increasing focus from researchers. Our study utilized bibliometric and visual analytical research methods to reveal the frontiers and hotspots of excellence in research in pediatric MASLD studies from 2004 to 2024. Through the construction of scientific maps and the visualization of research, the core publication is a clinical guideline as recommendations from the Expert Committee on NAFLD (ECON) and the North American Society of Pediatric Gastroenterology, Hepatology and Nutrition (NASPGHAN) (20). Currently, research in the field of pediatric MASLD study mainly focuses on the pathogenesis, pathological lesions in the liver, relationship with obesity, complications and outcomes, diagnosis, prevention, and management of pediatric MASLD. Insulin resistance, metabolic syndrome, steatohepatitis, hepatocellular carcinoma, cardiovascular risk, diabetes risk, diagnostic accuracy, lifestyle intervention, gut microbiome, probiotics, and MASLD are also the frontiers and focuses of pediatric MASLD studies.
